# Supplementary material for: Interpretation bias and contamination-based obsessive-compulsive symptoms influence emotional intensity related to disgust and fear
Source: PLoS One. 2020 Apr 30;15(4):e0232362. doi: 10.1371/journal.pone.0232362 (PMC7192464; doi:10.1371/journal.pone.0232362)
Supplement: S1 Appendix — (PDF) [file pone.0232362.s001.pdf]

1 Appendix

1.1 Text paragraphs

Table XX: All Text paragraphs used in Experiment 2

Category	Text paragraph	Disgust *M* (*SD*)	Fear *M* (*SD*)
Disgust-Neutral	Du gehst in eine öffentliche Toilette. Du bemerkst danach, dass Deine Schuhe nass geworden sind. [WC-nasse Schuhe]	**38.33 (23.96)**	2.29 (5.77)
	Du gehst mit einem Freund spazieren. Auf dem Rückweg bemerkt ihr einen unangenehmen Geruch. Du stellst fest, dass du in Hundekot getreten bist. [Hundekot]	**45.84 (32.71)**	3.61 (12.46)
	Du gehst mit Freunden ins Restaurant. Du bestellst dir einen Auflauf. Nachdem Du die Hälfte gegessen hast, findest Du ein Haar im Essen. [Haar im Essen]	**30.00 (27.09)**	0.15 (0.61)
	Du gehst Brötchen in einer Bäckerei kaufen. Da siehst Du, dass die Verkäuferin sich unübersehbar in der Nase kratzt. [Bäckernase]	**42.38 (30.92)**	3.44 (7.57)
	Du bestellst eine Pizza in einem Imbiss um die Ecke. Der Pizzabäcker ist erkältet und muss mehrmals niesen.[erkältete Pizza]	**48.59 (26.03)**	7.27 (21.44)
	Du sitzt in einer Vorlesung in der Uni. Beim Zuhören greifst Du an die Unterseite der Tischplatte. Dabei bleibt ein alter Kaugummi an Deinen Fingern kleben. [Kaugummi]	**35.53 (26.22)**	1.74 (5.25)
	Du gehst von einer Verabredung nach Hause. Zu spät merkst du, dass vor Deiner Haustür jemand erbrochen hat und trittst hinein. [Erbrochenes]	**57.5 (32.44)**	9.61 (20.58)
	Du unterhältst dich mit einer Dir noch unbekannten Person. Ihr unterhaltet euch nett. Mit der Zeit bemerkst Du einen unangenehmen Mundgeruch. [Mundgeruch]	**33.78 (22.57)**	2.89 (9.16)
Fear-Neutral	Du wartest auf einen guten Freund. Normalerweise ist er immer sehr pünktlich, aber heute ist er schon ein halbe Stunde zu spät. Sein Handy ist ausgeschaltet. [Vermisst]	1.73 (5.15)	**33.30 (30.86)**
	Mit einem großen Schreck wachst Du mitten in der Nacht auf,	2.84	**45.49**

	weil Du denkst ein Geräusch gehört zu haben, aber alles ist still. [Nachschreck]	(10.74)	**(26.83)**
	Du bis am Abend mit Freunden ausgegangen. Als Du gegen Mitternacht zu Hause ankommst siehst Du, dass die Wohnungstür offen steht. [Wohnungstür]	3.20 (12.09)	**70.43 (30.11)**
	Du gehst nach einer Feier nachts zu Fuß nach Hause. Ein kurzes Stück des Weges ist unbeleuchtet. [Unbeleuchtet]	0.54 (1.82)	**34.11 (30.55)**
	Du hast morgen ein Vorstellungsgespräch für einen Job, der Dir sehr gut gefällt. Du hast durch Bekannte erfahren, dass der Chef manchmal sehr streng sein kann. [Vorstellungsgespräch]	3.40 (15.68)	**36.44 (23.17)**
	Du bist schon eingeschlafen. Du wirst wach durch das mehrmalige Klingeln an deiner Wohnungstür. [Klingeln]	4.83 (20.35)	**41.38 (34.25)**
	Du bist auf dem Heimweg nach einer Geburtstagsfeier. Neben dir erscheint ein dunkler Schatten auf dem Boden. [Schatten]	5.19 (16.61)	**55.72 (32.69)**
	In einer Kneipe kommt ein wütender Mann auf Dich zu. [wütender Mann]	7.93 (16.37)	**45.47 (25.17)**
Disgust-Fear	Du bist auf einem Festival. Du gehst auf eine Dixi-Toilette. Währenddessen beginnen Personen an dem Toilettenhäuschen zu rütteln und es droht umzukippen. [Dixie-Toilette]	**70.15 (24.86)**	**60.67 (31.52)**
	Es ist dunkel und du befindest Dich auf dem Weg nachhause. Weil Du das Gefühl hast, dass Dich eine dunkle Gestalt verfolgt, achtest Du nicht auf den Weg. Auf einmal merkst Du, dass du in Hundekot getreten bist. [Verfolgt-Hundekot]	**31.57 (24.19)**	**37.46 (30.46)**
	Du besuchst eine öffentliche Toilette. Als du die Toilette verlassen möchtest, kannst Du die Tür nicht öffnen. Du bist eingesperrt in der öffentlichen Toilette. [Eingesperrt WC]	**20.48 (20.71)**	**9.93 (14.76)**
	Gleich musst du einen komplizierten und sehr wichtigen Vortrag halten. Da bemerkst Du, dass sich ein Pilz auf dem Tafelschwamm gebildet hat. [Vortrag-Schwamm]	**25.98 (26.27)**	**37.17 (28.42)**
	Du bist mit Freunden auf einer Feier. Da spricht Dich eine fremde Person an. Sie wirkt stark alkoholisiert und tritt Dir zu nahe. Währenddessen bemerkst Du einen großen nassen Flecken auf der Hose der fremden Person. [Fremder-nasse Hose]	**45.45 (28.01)**	**27.74 (29.13)**

	Du bist in einem Campingurlaub. An einem Tag beschließt Du außerhalb vom Zelt im Wald zu schlafen. Mitten in der Nacht wachst Du auf und spürst, dass sich etwas Glitschiges an deinen Beinen bewegt. [Schlafsack]	**58.41 (26.66)**	**52.65 (27.09)**
	Du fährst mit der Straßenbahn. Es wird sehr stark gebremst und Du musst Dich zusammen mit anderen Passagieren an einer Stange festhalten. Eure Hände berühren sich. [Straßenbahn]	**13.16 (23.96)**	**6.81 (17.33)**
	Du bist schwimmen in einem großen See. Auf einmal berührt Dich etwas am Fuß. [See]	**37.47 (30.28)**	**51.47 (29.77)**
Neutral	Du fährst nach der Uni auf einem Fahrrad nach Hause [Fahrrad]	0.00 (0.00)	0.95 (3.31)
	An einem schönen Sommertag fährst Du mit Freunden an den See. Ihr macht ein Picknick auf der Wiese. [See-Picknick]	0.43 (1.54)	0.30 (1.11)
	In den Semesterferien beschließt Du eine Freundin zu besuchen. [Sommerferien]	0.00 (0.00)	1.38 (4.99)
	Du ziehst in eine neue Wohnung ein. Um es Dir schön einzurichten, fährst du in ein Gartencenter um Dir Pflanzen zu kaufen. [Gartencenter]	0.38 (1.35)	0.75 (2.29)
